# Crimean–Congo Hemorrhagic Fever Virus: Advances in Vaccine Development

**DOI:** 10.1089/biores.2019.0057

**Published:** 2020-05-12

**Authors:** Thomas Tipih, Felicity Jane Burt

**Affiliations:** ^1^ Division of Virology, Faculty of Health Sciences, University of the Free State, Bloemfontein, South Africa.; ^2^ National Health Laboratory Service, Bloemfontein, South Africa.

**Keywords:** Crimean–Congo hemorrhagic fever virus, orthonairovirus, vaccine development, vaccine vector, virus-like replicon particles, recombinant protein

## Abstract

Crimean–Congo hemorrhagic fever (CCHF) is a severe human disease with mortality rates of up to 30%. The disease is widespread in Africa, Asia, the Middle East and Eastern Europe. The last few years have seen disease emergence in Spain for the first time and disease re-emergence in other regions of the world after periods of inactivity. Factors, such as climate change, movement of infected ticks, animals, and changes in human activity, are likely to broaden endemic foci. There are therefore concerns that CCHF might emerge in currently nonendemic regions. The absence of approved vaccines or therapies heightens these concerns; thus Crimean–Congo hemorrhagic fever virus (CCHFV) is listed by the World Health Organization as a priority organism. However, the current sporadic nature of CCHF cases may call for targeted vaccination of risk groups as opposed to mass vaccinations. CCHF vaccine development has accelerated in recent years, partly because of the discovery of CCHF animal models. In this review, we discuss CCHF risk groups who are most likely to benefit from vaccine development, the merits and demerits of available CCHF animal models, and the various approaches which have been explored for CCHF vaccine development. Lastly, we present concluding remarks and research areas which can be further explored to enhance the available CCHFV vaccine data.

## Introduction

### Background

Crimean–Congo hemorrhagic fever virus (CCHFV) is exclusively associated with a virulent disease in humans. In the absence of approved therapeutics or vaccines against the virus, treatment is predominantly supportive. CCHFV possesses a trisegmented negative-sense RNA genome and is classified within the *Orthonairovirus* genus of the *Nairoviridae* family. Crimean–Congo hemorrhagic fever (CCHF) was medically recognized in 1944 in the wake of an outbreak involving military personnel stationed in the Crimean peninsula, and the medical condition was named Crimean hemorrhagic fever (CHF).^1^ A viral etiology and a tick-borne origin for CHF were proposed after *Hyalomma marginatum* tick filtrates produced the disease in human volunteers and individuals with psychiatric disorders.^2^ Following the Crimean peninsula outbreak, numerous epidemics of related disease conditions were described in Central Asia, Bulgaria, and the Soviet Union.^1^ Meanwhile, Dr. Courtois from the Belgian Congo isolated a virus from a febrile teenage boy using newborn mice in 1956, and the virus was designated Congo virus strain V3011.^1^ The causative agent of CHF was isolated in 1967 in newborn mice after intracerebral inoculation.^3,4^ Characterization studies of agents responsible for global tick-borne diseases, at the Yale Arbovirus Research Unit, established that the agent causing CHF was antigenically similar to Congo virus strain V3011.^5^ The names were subsequently combined and the virus named CCHFV.^1^

CCHFV is sustained in an enzootic cycle encompassing ticks and several vertebrate animals with humans regarded as dead-end hosts. Sources of human infections include bite from an infected tick, close contact with blood or tissue from diseased animals, and CCHF patients. Animals do not display symptoms, but disease in humans progresses through four phases: incubation, prehemorrhagic, hemorrhagic, and convalescence.^1^ Incubation period depends on the route of transmission; 1–3 days, up to 9 days for tick transmission while after exposure to infected blood or tissues incubation period is mostly 5–6 days and reach up to 13 days.^1,6^

### Geographic distribution

**FIG. 1.**
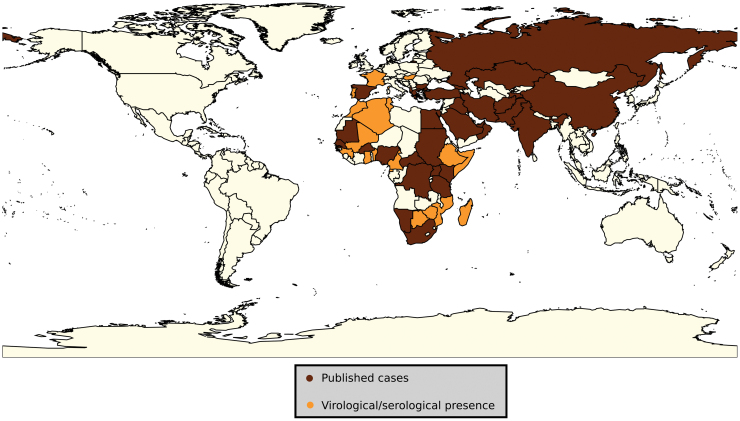
Global geographic summary of countries with reported CCHF cases, serological evidence and presence of CCHFV in ticks.^1,7–48^ CCHF, Crimean–Congo hemorrhagic fever; CCHFV, Crimean–Congo hemorrhagic fever virus.

CCHFV is an emerging and re-emerging virus with extensive geographical distribution, as shown in Figure 1. The virus has a constant presence in Africa, the Middle East, Asia, and Eastern Europe. African countries from which CCHF has been reported include Burkina Faso, Central Africa Republic, Democratic Republic of Congo, Egypt, Kenya, Mauritania, Namibia, Nigeria, South Africa, Senegal, Sudan, Tanzania, and Uganda^1,7–16^ and countries yet to report CCHF but with evidence of viral circulation either from serological surveys or CCHFV isolation from ticks include Algeria, Benin, Cameroon, Equatorial Guinea, Ethiopia, Ghana, Guinea, Mali, Madagascar, Morocco, Mozambique, Niger, Somalia, Tunisia, and Zimbabwe.^1,17–30^ The presence of CCHFV in Somalia was suggested after evidence of the CCHFV in *Hyalomma* ticks obtained from Somali cattle and sheep exported to the United Arab Emirates.^28^ In the Middle East, the disease has been described in Iran, Iraq, Saudi Arabia, Oman, and the United Arab Emirates.^31–35^ East European countries with described CCHF disease include Albania, Bulgaria, Greece, Kosovo, Turkey, Georgia, and Russia.^36–42^ Portugal, Hungary, France, and Romania^1,43–45^ are at risk of CCHF based on serological evidence of viral circulation. Asian countries with described CCHF disease include Afghanistan, Kazakhstan, Pakistan, China, India, Tajikstan, and Uzibekistan.^1,46–49^

CCHF cases have over the past few years been described in new regions while the disease has re-emerged in some countries after periods of inactivity. CCHF was first described in Turkey in the year 2000, and the country has the highest incidence of CCHF cases per annum with a mortality rate slightly below 5%.^50^ Over 1660 cases were reported by Russian Pro-Med between 2005 and 2017.^51^ The first human case in Iran was reported in 1999, and an increase in incidence has been observed.^52,53^ A nosocomial outbreak of CCHF at a hospital in India in 2011 stands as the principal case in the country^49^ and from there on, sporadic cases have been reported.^54^ The first recorded CCHF cases in Spain were described in 2016 from an adult male (an index case) who most likely acquired infection through a tick bite and a nosocomially acquired infection by a health professional who nursed the index case.^55^ Even though CCHF has not been described in some countries, the presence of CCHFV in ticks and CCHFV-specific antibodies in wild and domesticated ruminants have been demonstrated.^56^ CCHFV endemic foci are expected to broaden in the face of climate change,^57^ human case movement as well as the movement of animals infested with a tick.^58^ However, in some regions, the observed increase in reported cases could be a product of improved awareness and diagnostic capacity. CCHFV poses a zoonotic risk with public health implications and as such CCHF is a notifiable disease. Nonetheless, with the exception of Turkey reporting more than 50 cases per year,^59^ CCHF cases are currently sporadic in endemic zones.

### CCHF vaccine target population

Considering that CCHF cases are sporadic, global mass vaccinations are unlikely, thus targeting vaccinations would be ideal. People residing in endemic areas who are prone to tick bites, especially *Hyalomma* ticks, are at risk of acquiring CCHF.^60^ Development of an efficacious vaccine will prevent infections and possible mortality from the disease in risk groups. CCHF vaccine target population would include farmers dealing with agriculture or animal husbandry. Even though livestock does not develop CCHF, they present with transient viremia. Contact with blood or tissues from infected livestock and bites from infected ticks have been reported as transmitting CCHFV, resulting in outbreaks in farming communities.^61^ Besides farmers, individuals participating in religious activities such as the Muslim Eid-ul Azha who get exposed to livestock blood and tissue during animal slaughter are at an increased risk of CCHF.^62^ Veterinarians and abattoir workers stand a high chance of occupational exposure to tick bites or contact with viremic animal blood and thus constitute another risk group. Outdoor activities such as hiking and camping as well as some rural lifestyles where people live in close proximity to livestock pose as risk factors for tick bites and contact with infected animals.^63,64^

Nosocomial transmission constitutes a major route of CCHFV spread accounting for a large proportion of global CCHF cases,^65^ often with higher case fatality rates compared with tick bite infections.^66^ Nosocomial outbreaks often serve as an indicator of unrecognized infections in the general population and seroepidemiological surveys carried in the wake of nosocomial outbreaks reveal prior presence of the virus in the community. Human serum CCHFV titers are high (10^8^–10^10^ copies/mL) during the prehemorrhagic stage, especially in fatal cases^67^ and the virus has been detected in urine and saliva.^68^ The high CCHFV titers put medical professionals in contact with patients at risk and the nonspecific symptoms early in the course of disease exacerbates the risk. Laboratory personnel handling live virus comprise another risk group who could be a target for the CCHF vaccine. Laboratory-acquired infections arising from handling patient samples^49,69–71^ and during CCHFV research activities^70,72^ have been reported. Besides humans, livestock could be vaccinated to prevent viremia in animals and subsequent transmission to humans. Vaccinating livestock also prevents them from serving as amplifying hosts although small mammals are considered principal amplifying hosts.

### CCHF animal models

Animal models for CCHF have recently been described. Previously, besides humans, the only other vertebrate known to be susceptible to CCHFV were newborn mice and rats. The immature immune systems of these infant rodents meant these animals could not serve as models. CCHF infections in immunocompetent animals result in transient viremia and absence of noticeable symptoms.^73,74^ Experimental infection of knockout mice with CCHFV displayed some disease signs and physiological changes, which parallel findings in humans, although differences are found in rapidity of disease onset and level of lethality. The first animal models described lacked signaling either to all the three types of interferons (STAT-1^−/−^)^75^ or type 1 interferon (IFNAR^−/−^).^76^ Besides the knockout mice, a mouse model whose type 1 interferon system is temporarily suppressed IS has been described.^77^ The knockout models and the (IS) model are permissive to infection, succumbing to CCHFV infection within 5 days. STAT-1 knockout mice present with leukopenia, thrombocytopenia, elevated levels of serum hepatic alanine aminotransferase, and proinflammatory cytokines.^75^ CCHFV RNA is widespread in tissues of STAT-1^−/−^ and IFNAR^−/−^ mouse models.^75,76^ Additionally, a humanized mouse model was prepared by injecting NSG-SGM3 mice with CD34^+^ human stem cells.^78^ The humanized model displayed different disease patterns when inoculated with CCHF strain from Oman and Turkey. Lethal outcomes and neurological disease were only observed with the Turkish strain.^78^ A nonhuman primate model was described in 2018. *Cynomolgus macaque* infected with the Kosova Hoti CCHFV strain developed disease patterns and outcomes characteristic of CCHF human cases.^79^ In an independent comparative investigation in *Cynomolgus macaque*, animals infected with the CCHFV Afghanistan strain (Afg09-2990) and the Kosova Hoti CCHFV strain developed a clinical picture, laboratory clinical chemistry, and hematological parameters, as well as serum cytokine levels commonly seen in humans.^80^ However, all the 12 animals recovered, unlike the infection studies involving the Kosova Hoti strain, in which only 25% (1/4) of the animals did not meet euthanasia criteria. The nonhuman primate model has provided evidence regarding the ability of CCHFV to replicate and persist in the testes of monkeys, opening the possibility of sexual transmission.^81^

## Vaccines Against CCHF

### Bulgarian vaccine

Even though there is not a globally recognized vaccine for CCHFV, there is a vaccine that has been in use in Bulgaria since 1974. The Bulgarian vaccine originated in the Union of Soviet Socialist Republics (USSR). This is an inactivated vaccine prepared from brain tissue of CCHFV infected newborn mice. Inactivation of the CCHFV particles was brought about by a combination of chloroform and heat treatment. The vaccine is administered subcutaneously multiple times in risk groups who are more than 16 years old. Between 1974 and 1996, there was a precipitous drop in cases (from 1105 to 279) reported to the Bulgarian Ministry of Health and fewer than 20 cases reported per annum after 1996.^82^ Remarkably, there were no reported cases from military and laboratory personnel who were vaccinated.^82^ Nevertheless, the decrease in reported Bulgarian cases could have been independent of vaccine efficacy but a product of other factors. The observed reduction could have been attributed to a change in CCHFV epidemiology and ecology in the absence of deliberate intervention.^83^ Increased CCHFV awareness could have led to behavior change thus reduced tick exposure or a different case definition and reporting following the introduction of vaccine.^83^

Although the mouse brain-derived vaccine has been in use in Bulgaria, the vaccine is not a viable option for widespread global use largely due to safety concerns and lack of efficacy trials. Propagating CCHFV in brain tissue of newborn mice requires biosafety level 4 (BSL-4) facilities. International approval of this vaccine is unlikely because of safety concerns surrounding the mouse neural tissue content, which has potential to cause autoimmune and allergic responses^84^ and the requirement for high containment facilities for propagation. Furthermore, the Bulgarian vaccine requires multiple immunizations with vaccinations every 5 years to preserve immunity. Individuals below the age of 16 do not qualify to receive the vaccine and leaves a fraction of the population without immunity to the virus. Apart from that, the efficacy of the Bulgarian vaccine is yet to be demonstrated in clinical trials.

## CCHFV Protein Targets for Vaccine Development

Recent, CCHF vaccine development has focused on the viral glycoproteins and the nucleoprotein (NP) even though the immune correlates of protection are yet to be described. The investigated CCHFV proteins are recombinant proteins produced either *in vitro* or *in vivo*. *In vitro* proteins were produced in cell cultures, purified, and vaccinated into animal models while *in vivo* protein production utilized vectors, which were used to deliver genes encoding CCHFV antigens facilitating endogenous protein production. *In situ* antigen generation is desirable since proteins acquire post-translational modifications similar to natural infections.^85^

### Nucleoprotein

The role of the CCHFV NP in protection against infection and clearance of viruses is not known. However, the NP possesses features which make the antigen ideal for CCHFV vaccine development. The protein is produced in large amounts during infection and is highly immunogenic containing B and T cell epitopes.^86,87^ Besides that, the NP amino acid sequence shows the least variation^88^ thus, an NP-based vaccine is expected to offer protection against the diverse CCHFV strains. Recently, complete protection in knockout mice against CCHFV challenge infection has been reported after vaccination with NP-based vaccines using different expression systems.^89–90^ Even though the NP is an internal protein and is not expected to induce neutralizing antibodies, the NP is released from infected cells^91^ thus can interact with antibodies forming immune complexes capable of antiviral activity.^92^

### Glycoproteins

The CCHFV M segment encodes a polyprotein glycoprotein precursor (GPC), which is post-translationally processed to intermediate glycoproteins (pre-Gn and pre-Gc). Further processing of the intermediary glycoproteins yields envelope glycoproteins Gn and Gc, nonstructural M protein (NS_M_) as well as secreted nonstructural proteins (GP160, GP85, and GP38) and mucin-like domain.^93–95^ The secreted GP38 has been demonstrated to localize to viral and cellular membranes of cells expressing the M segment.^96^ The glycoproteins Gn and Gc have been largely considered as the antigen of choice for the CCHFV vaccine chiefly because they are located on the surface of virus particles and hence considered responsible for inducing neutralizing antibodies. To this end, monoclonal neutralizing antibodies against the pre-Gn and Gc glycoproteins were described.^97^ Recently all the neutralizing antibodies previously reported to target the pre-Gn interacted with the GP38 and none interacted with the Gn.^96^ mAb-13G8, a GP38 specific non-neutralizing monoclonal antibody protected IFNAR^−/−^ mice against lethal infection, whereas Gc-specific neutralizing antibodies could not offer protection^96^ despite demonstrating *in vitro* virus neutralization.^97^ Passive administration of mAb-13G8 (homologous to the IbAr 10200 CCHFV strain) in IFNAR^−/−^ mice displayed limited protection against a heterologous CCHFV Afg09-2990 strain.^96^ The diversity of the M segment, especially the region encoding the nonstructural proteins, has long been suspected to impact crossreactivity and ultimately, the neutralization ability against heterologous strains. One factor contributing to the diversity of the M segment is genetic reassortment. The consequence of reassortment on viral aspects, such as replication, transmission, virulence, and immunogenicity is yet to be fully investigated.

### CCHF vaccine candidates

The search for a CCHF vaccine has accelerated in recent years, and this has been partly attributed to the discovery of animal models for CCHF. Before the recognition of the animal models, there have been few attempts described in literature and vaccine efficacy studies were not performed. Investigated CCHF vaccine approaches include subunit antigen preparations, genetically modified plants, as well as DNA and viral vectors expressing CCHFV antigens, transcriptionally competent virus-like particles (VLPs), messenger RNA (mRNA) vaccine, and inactivated whole CCHFV particles.^98–105^ A summary of the investigated CCHFV vaccine approaches and outcomes is outlined below.

#### Subunit vaccines

Using insect expression technology, the ectodomains of the CCHFV envelope glycoproteins Gn and Gc from the IbAr 10200 strain were expressed in *Drosophila* Schneider 2 (S2) cells and purified. The Gn and Gc proteins formulated with the Sigma Adjuvant system separately elicited antibodies with neutralizing capacity *in vitro* after intraperitoneal vaccination in STAT129 mice using a prime and boost strategy (Table 1). However, the elicited *in vitro* neutralizing antibodies could not offer protection following subcutaneous CCHFV challenge infection.^98^

**Table 1.** Approaches in the Development of CCHFV Vaccines

Vaccine type	CCHFV antigen^a^	Mouse model	Dose^b^	Antibody response	T cell response	Challenge^c^	Efficacy, % survival	Reference
Recombinant protein	Gc-e ectodomain (adjuvanted) Gn ectodomain (adjuvanted) Gc-eΔ ectodomain (adjuvanted)	STAT-1^−/−^ STAT-1^−/−^ STAT-1^−/−^	1.4 μg (i.p.) day 0 and 21 15 μg (i.p.) day 0 and 21 7.5 μg (i.p.) day 0 and 21	Yes^d^ Yes^d^ Yes^d^	NT NT NT	100 pfu CCHFV IbAr 10200 strain (s.c.) day 42 100 pfu CCHFV IbAr 10200 strain (s.c.) day 42 100 pfu CCHFV IbAr 10200 strain (s.c.) day 42	0% 0% 0%	^98^ ^98^ ^98^
Transgenic plants	Gn and Gc (Iranian strain)	BALB/c BALB/c BALB/c BALB/c BALB/c	Fed leaves 10 μg 5 × (1-week intervals) Fed roots 10 μg 5 × (1-week intervals) Fed leaves 10 μg 4 × (1-week intervals) and injected 5 μg Gn/Gc Fed roots 10 μg 4 × (1-week intervals) and injected 5 μg Gn/Gc Bulgarian vaccine, injected four doses at 2-week intervals (s.c.)	Yes^e^ Yes^e^ Yes^e^ Yes^e^ Yes^e^	NT NT NT NT NT	NT NT NT NT NT	NT NT NT NT NT	^99^ ^99^ ^99^ ^99^ ^99^
Virus-like replicon particles	GPC, L, and NP (IbAr 10200 L, NP, and Oman-1998 GPC ) GPC, L, and NP (IbAr 10200 L, NP, and Oman-1998 GPC) GPC, L, and NP (IbAr 10200 L, NP, and Oman-1998 GPC) Gn, Gc, and NP	IFNAR^−/−^ IFNAR^−/−^ IFNAR^−/−^ IFNAR^−/−^ IFNAR^−/−^	High dose (10^5^ TCID_50_) (s.c.)^f^ Low dose (10^3^ TCID_50_) (s.c.)^f^ 10^5^ TCID_50_ (s.c.)^f^ 10^5^ TCID_50_ (s.c.)^f^ 10^6^ VLPs/mouse (i.p.) day 0, 28, and 49	Yes^e^ Yes^e^ Yes^e^ Yes^e^ Yes^d^	NT NT NT NT Yes	100 TCID_50_ recombinant CCHFV-IbAr 10200 (s.c.) day 32 100 TCID_50_ recombinant CCHFV-IbAr 10200 (s.c.) day 32 100 TCID_50_ CCHFV Oman-199723179 (s.c.) day 28 100 TCID_50_ CCHFV Turkey-200406546 (s.c.) day 28 400 FFU CCHFV strain IbAr 10200 (i.p.) day 91	100% 78% 100% 100% 40%	^109^ ^109^ ^110^ ^110^ ^100^
DNA	GPC Gn, Gc, and NP GPC GPC NP (Ank-2 strain) NP (Ank-2 strain) NP (Ank-2 strain)	BALB/c BALB/c IFNAR^−/−^ IFNAR^−/−^ IS C57BL/6 BALB/c and IFNAR^−/−^ BALB/c and IFNAR^−/−^ BALB/c and IFNAR^−/−^	10 μg (g.g.) day 0, 28, 56, and 84^g^ 2.5 μg (g.g.) day 0, 28, 56, and 84^h^ 50 μg (i.d.) day 0, 28, and 49 25 μg (i.m.) day 0, 21, and 42 25 μg (i.m.) day 0, 21, and 42 50 μg (i.m.) day 0 and 14 pV-N13 (50 μg) (i.m.) day 0 and 14 pV-N13 (40 μg) + pCD24 (10 μg) (i.m.) day 0 and 14	Yes^d^ Yes^d^ Yes^d^ Yes^d^ Yes^d^ Yes^i^ Yes^i^ Yes^i^	NT NT Yes NT NT Yes Yes Yes	NT NT 400 FFU CCHFV strain IbAr 10200 (i.p.) day 91 100 pfu CCHFV strain IbAr 10200 (i.p.) day 70 100 pfu CCHFV strain IbAr 10200 (i.p.) day 70 1000 TCID_50_ of Ank-2 strain (i.p.) day 28 1000 TCID_50_ of Ank-2 strain (i.p.) day 28 1000 TCID_50_ of Ank-2 strain (i.p.) day 28	NT NT 100% 71% 60% 75% ^*^50% 100% 100%	^113^ ^113^ ^100^ ^77^ ^77^ ^90^ ^89^ ^89^
mRNA	NP	IFNα/βR^−/−^	25 μg (i.m.)^f^ 25 μg (i.m.) day 0, day 14	Yes^i^ Yes^i^	Yes Yes	1000 TCID_50_ of Ank-2 strain (i.p.) day 42 1000 TCID_50_ of Ank-2 strain (i.p.) day 56	50% 100%	^104^ ^104^
MVA vector Recombinant adenovirus type 5 Recombinant vesicular stomatitis virus Recombinant BoHV-4 Recombinant adenovirus type 5 Formalin inactivated vaccine	GPC GPC NP NP NP (3010 strain) NP (3010 strain) NP NP GPC GPC NP (Ank-2 strain) NP (Ank-2 strain) Whole virus particle (Turkey-Kelkit06 strain)	IFNα/βR^−/−^ 129Sv/Ev IFNα/βR^−/−^ 129Sv/Ev IFNα/βR^-/^ 129Sv/Ev IFNAR^−/−^ IFNAR^−/−^ STAT-1^−/−^ STAT-1^−/−^ BALB/c and IFNAR^−/−^ BALB/c and IFNAR^−/−^ IFNAR^−/−^ IFNAR^−/−^ IFNAR^−/−^	10^7^ pfu MVA-GP (i.m.) day 0 and 14 10^7^ pfu MVA-GP (i.m.) day 0 and 14 10^7^ pfu MVA-NP10200 (i.m.) day 0 and 14 10^7^ pfu MVA-NP10200 (i.m.) day 0 and 14 10^7^ pfu MVA-NP3010 (i.m.) day 0 and 14 10^7^ pfu MVA-NP3010 (i.m.) day 0 and 14 1.25 × 10^7^ ifu (i.m.)^f^ 1.25 × 10^7^ IFU (i.m.) day 0 and 10^8^ IFU (i.n.) day 28 10^7^ pfu (i.p.)^f^ 10^7^ pfu (i.p.) day 0 and 14 100 TCID_50_ (i.p.) day 0 and 14 100 TCID_50_ (i.p.) day 0 and 14 5 μg (i.p.) day 0, 21 and 42 20 μg (i.p.) day 0, 21 and 42 40 μg (i.p.) day 0, 21 and 42	Yes^e^ Yes^e^ Yes^e^ Yes^e^ Yes^e^ Yes^e^ NT Yes^e^ Yes^d^ Yes^d^ Yes^i^ Yes^i^ Yes^d^ Yes^d^ Yes^d^	Yes Yes Yes Yes Yes Yes NT NT NT NT Yes Yes NT NT NT	200 TCID_50_ CCHFV virus strain IbAr 10200 (i.d.) day 28 Not challenged 200 TCID_50_ CCHF virus strain IbAr 10200 (i.d.) day 28 Not challenged Not challenged Not challenged 50 TCID_50_ CCHFV strain IbAr 10200 (s.c.) day 28 50 TCID_50_ CCHFV strain IbAr 10200 (s.c.) day 56 50 pfu of CCHFV strain Turkey200406546 (i.p.) day 35 50 pfu of CCHFV strain Turkey200406546 (i.p.) day 35 1000 TCID_50_ of Ank-2 strain (i.p.) day 28 1000 TCID_50_ of Ank-2 strain (i.p.) day 28 1000 PPFU Turkey-Kelkit06 strain (i.p.) day 56 1000 PPFU Turkey-Kelkit06 strain (i.p.) day 56 1000 PPFU Turkey-Kelkit06 strain (i.p.) day 56	100% N/A 0% N/A N/A N/A 33% 78% 100% 100% 100% 75%^j^ 100% 50%^j^ 60% 80% 80%	^101^ ^101^ ^102^ ^102^ ^102^ ^102^ ^103^ ^103^ ^116^ ^116^ ^90^ ^90^ ^105^ ^105^ ^105^

^a^ All vaccine candidates were based on IbAr 10200 strain unless otherwise stated.

^b^ Vaccine dose, timing, and route of inoculation, i.d.; i.p.; i.m.; i.n.; s.c.; g.g.

^c^ CCHFV challenge strain, dose, route, and timing.

^d^ Neutralizing antibodies *in vitro*.

^e^ Antibody ability to neutralize *in vitro* not assessed.

^f^ Single-dose administered.

^g^ Mice immunized with 10 μg of CCHF DNA vaccine.

^h^ Mice immunized with 2.5 μg of each of Rift Valley fever virus, CCHF, Hantaan virus and tick-borne encephalitis virus DNA vaccine.

^i^ Non-neutralizing antibodies *in vitro*.

^j^ Antibody passive and T cell adoptive transfer experiment.

BoHV-4, bovine herpesvirus type 4; Crimean–Congo hemorrhagic fever; CCHFV, Crimean–Congo hemorrhagic fever virus; ffu, focus-forming units; g.g., gene gun; GPC, glycoprotein precursor; i.d., intradermal; ifu, infectious units; i.m., intramuscular; i.n., intranasal; i.p., intraperitoneal; IFU, infectious units; IS, transiently suppressed type 1 interferon system; L, RNA-dependent RNA polymerase; mRNA, messenger RNA; MVA, modified Vaccinia Ankara virus; NP, nucleoprotein; NT, not tested; pfu, plaque-forming unit; PPFU, pseudo plaque-forming unit; s.c., subcutaneous.

#### Plant-based vaccines

Genetically engineered plants can express foreign antigen for vaccine development purposes. Approaches for foreign gene expression in transgenic plants include stable transgenic plants, use of viral vectors for transient expression, and the chloroplast expression system.^106,107^ The nonrequirement of the cold chain for the recombinant proteins and production of abundant biologically active proteins relatively inexpensively makes plant-based vaccines appealing especially for developing countries.

Vertebrate animals, particularly domestic animals, are significant in CCHFV transmission cycle. Reducing viral amplification in vertebrates could decrease CCHFV transmission to humans. Genetically modified tobacco plants expressing the envelope glycoproteins Gn and Gc from an Iranian strain were fed to BALB/c mice (Table 1). The Gn and Gc glycoproteins were genetically engineered to form one reading frame. Immunized mice elicited CCHFV-specific anti-Gn/Gc IgG and IgA antibodies in serum and fecal material. End boost groups induced higher endpoint antibody titers (1:32768) compared with the fed groups (1:256). Interestingly, fecal pellets had higher IgA endpoint titer (1:512) compared with serum (1:256).^99^ The neutralizing potential of the antibodies was not assessed, and challenge studies were not performed.

#### Virus-like replicon particles

Using reverse genetics, a transcriptionally active virus-like particle (tc-VLP) system, has been developed.^108^ Structurally, the VLPs consists of a genome like a reporter encapsidated by the CCHFV NP and L protein enclosed in a membrane displaying Gn and Gc proteins on the surface. Thus, VLPs have morphology and protein antigenicity resembling native CCHF viruses. Vaccinating three times intraperitoneally with the tc-VLP displaying the envelope glycoproteins (Gn and Gc) from the CCHFV IbAr 10200 strain on their surface was accompanied by a strong induction of *in vitro* neutralizing antibodies in an IFNAR^−/−^ mice model, which protected 40% of the challenged mice^100^ (Table 1). Cytokine analysis before challenge infection demonstrated induction of Th2-type immunity, whereas postchallenge cytokine analysis could not be performed.^100^

A virus-like replicon particle (VRP) vaccine candidate based on IbAr 10200 strain but with the GPC sequence from the Oman-1998 strain provided complete protection against lethal challenge following a single high dose (10^5^ TCID_50_ of VRP) subcutaneous vaccination in IFNAR^−/−^ mouse model.^109^ In contrast, a low dose (10^3^ TCID_50_ of VRP) vaccination protected seven out of nine mice (Table 1). In a related study, the VRP candidate vaccine provided complete protection against challenge with each of the CCHFV IbAr 10200 strain, the CCHFV-Turkey strain, and the CCHFV-Oman-97 strain^110^ (Table 1).

#### DNA vaccines

DNA vaccines do provide an attractive alternative for emerging and re-emerging pathogens such as CCHFV. DNA vaccines are temperature stable and can be designed to incorporate specific immunogenic viral proteins desired for immunization. Depending on the immune correlates of protection, genetic vaccines can be tailored to raise either type 1 T-helper (Th) or type 2 Th cell responses.^111^ DNA immunization also allows for the differentiation between natural infections and vaccine-induced responses since specific antigens are selected. The mechanisms of inducing cytotoxic T lymphocyte-mediated adaptive immunity are similar for DNA vaccines and live attenuated vaccines, but the risk of reverting to virulence associated with the latter is eliminated in DNA vaccines.^112^ A DNA-based vector designed to deliver the GPC of the IbAr 10200 CCHFV strain was first described in 2006. The CCHFV vaccine construct was either delivered three times individually or coadministered with DNA vectored vaccine constructs for Hantaan and Rift Valley fever viruses encoding the GPC and tick-borne encephalitis virus encoding the premembrane and envelope genes (Table 1). Fifty percent of the vaccinated BALB/c mice either with the CCHFV DNA vaccine or combined with other vaccine constructs developed *in vitro* neutralizing antibodies.^113^ Induction of cell-mediated immune responses was not evaluated and challenge studies were not performed. Intradermal immunization (three times) of a DNA vector encoding the mature CCHFV envelope glycoproteins (Gn and Gc) and the NP of the IbAr 10200 strain, elicited antibody and T cell immune responses, which protected IFNAR^−/−^ mice against lethal CCHFV challenge^100^ (Table 1). Curiously, mice which received a VLP construct in the same study presented with higher *in vitro* neutralizing antibodies compared with the CCHFV DNA vaccine but protection was partial. These findings imply that neutralizing antibodies are not the sole correlate of CCHFV protection.

Vaccine immunogenicity and efficacy studies in IFNAR^−/−^ and interferon receptor antibody transiently suppressed (IS) mouse models were compared after intramuscular electroporation of a DNA expression vector encoding the entire GPC of the IbAr 10200 CCHFV strain^77^ (Table 1). Intraperitoneal (i.p.) route for the challenge was chosen based on previous observations that the i.p. route displayed the most rapid disease onset compared with the intramuscular, intranasal, and subcutaneous routes.^114^ Antibody responses in the two mouse models reflected a predominant Th1 response and the IS mouse model had a significantly lower Th1/Th2 ratio indicating balanced antibody responses with the immunocompetent mice.^77^ Although a higher survival rate after the lethal challenge was observed in the IFNAR^−/−^ model, with 71.4% (5 out 7 animals) compared with the IS model, with 60% (6 out 10 animals), this was not statistically different. Significantly, complete protection was not achieved in both mouse models.^77^

Vaccinating IFNAR^−/−^ mice twice with a DNA vector encoding the complete NP of the CCHFV Turkey-Kelkit06 or codelivery of the DNA vectors encoding the complete NP of the CCHFV Turkey-Kelkit06 and the cluster differentiation 24 (CD24), protected animals from lethal challenge infection^89^ (Table 1). Codelivery of CD24 and NP significantly enhanced induction of Th1 and Th2 cytokines as well as antibody responses, although the elicited antibodies lacked neutralization ability. Intramuscular administration of a DNA vector encoding the complete NP gene of the CCHFV Turkey-Kelkit06 produced 75% protection from lethal challenge infection, whereas a 50% survival rate was observed in the antibody passive transfer and T cell adoptive transfer experiment^90^ (Table 1). Despite the induction of high antibody titers and protective efficacy in animal models, elicited NP-specific antibodies in both studies could not neutralize CCHFV *in vitro*. Absence of neutralizing antibodies should not diminish the NP as a potential vaccine candidate because non-neutralizing antibodies can be protective by promoting phagocytosis, complement, or antibody-dependent cellular cytotoxicity.^115^

#### mRNA vaccine

A naked conventional CCHF mRNA vaccine has been described. The vaccine expresses the NP of the CCHF Ank-2 strain flanked by a 5′ cap (antireverse cap analog), 3′-polyA tail, 3′ and 5′ untranslated regions to enhance stability and translation of the construct. Intramuscular vaccination of IFNAR^−/−^ using a prime boost approach provided 100% protection following viral challenge while a single dose of the vaccine construct conferred 50% protection^104^ (Table 1).

#### Viral vectored vaccines

Recombinant viruses have been extensively investigated as vectors in gene therapy and gene delivery for vaccine development. Viral expression systems explored for CCHF vaccine development comprise the modified Vaccinia Ankara virus (MVA),^101,102^ recombinant vesicular stomatitis virus (rVSV),^116^ recombinant adenovirus type 5 (AdV-5),^90,103^ and recombinant bovine herpesvirus type 4 (BoHV-4).^90^ The MVA platform was used to deliver the GPC and the NP of the IbAr 10200 CCHFV strain. The N-termini of the GPC and NP were fused to the human tissue plasminogen activator leader sequence, whereas a V5 epitope tag was fused to the C-termini. An mH5 promoter was selected to drive gene transcription. The MVA-based vaccine constructs were administered two times intramuscularly (Table 1) and the construct designed to encode the GPC-induced *in vitro* neutralizing antibodies and T cell responses and complete protection in IFNAR^−/−^ mice^101^ after intradermal lethal challenge. Although the MVA-delivered NP induced humoral and cellular immune responses in vaccinated mice, the immune responses could not protect animals from lethal challenge infection.^102^ A replication-competent recombinant VSV encoding the CCHFV GPC gene of the IbAr 10200 CCHFV strain yielded 100% protection in a STAT-1^−/−^ mouse model following single intraperitoneal immunization, while a replication-deficient VSV construct did not confer protection from intraperitoneally administered lethal virus challenge^116^ (Table 1). The replication-competent construct developed nonsynonymous single nucleotide polymorphisms (SNPs), and two of these SNPs were nonsense mutations, which resulted in the truncation of part of the C-terminal tail of the Gc protein. Interestingly, although 100% protection was observed with the prime and boost group, the prime group elicited higher IgG and *in vitro* neutralizing antibody titers compared with the boost group at the endpoint. The clinical data and immunohistochemistry analysis of the spleen and liver of study animals suggested higher CCHFV replication in the prime group compared with the boost group thus viral challenge may have served as a heterologous booster for the prime group.^116^ A CCHFV NP-based candidate vaccine based on the human adenovirus 5 encoding the NP of the CCHFV strain IbAr 10200 partially protected IFNAR^−/−^ mice against virus challenge^103^ (Table 1). A prime-boost strategy improved protection and resulted in reduced clinical signs compared with single-dose vaccination. IFNAR^−/−^ mice immunized intraperitoneally with a recombinant AdV-5 encoding the NP from the Turkey-Kelkit06 strain survived challenge with CCHFV Ank-2 strain, and half of the mice survived a lethal challenge in the antibody passive transfer and T cell adoptive transfer experiment^90^ (Table 1).

In the face of safety challenges and antivector immunity posed by commonly used viral expression platforms, there is a need to explore new platforms. BoHV-4 possesses features such as a less complex genome compared with other herpesviruses coupled by large package size, easy growth in cell culture, limited or no pathogenicity or oncogenicity,^117^ and availability of an animal model (rabbit),^118^ which makes it a good candidate. A BoHV-4 vector encoding the full-length NP of the CCHFV Turkey-Kelkit06 strain utilizing a prime and boost strategy provided complete protection of IFNAR^−/−^ mice against lethal challenge infection, and partial protection was observed in the antibody passive transfer and T cell adoptive transfer experiment^90^ (Table 1).

#### Inactivated vaccines

The CCHFV Turkey-Kelkit06 strain was propagated in cell culture, harvested, and inactivated by formaldehyde to prepare an inactivated vaccine. The vaccine was mixed with the Imject Alum adjuvant and delivered intraperitoneally using a prime, boost, and boost strategy^105^ in IFNAR^−/−^ mice (Table 1). Vaccine doses of 5, 20, and 40 μg were investigated. In immunogenicity studies, the 5 μg dose group induced the lowest levels of *in vitro* neutralizing antibody titers and the increase in antibody titer was dose dependent. Despite differences in the levels of neutralizing antibodies, similar survival rate (80%) was observed with the 20 and 40 μg dose groups in IFNAR^−/−^ mice following lethal challenge infection. The effect of the vaccine on the T cell immune response was not evaluated.

## Future Directions and Concluding Remarks

Interferon-deficient mice and the *Cynomolgus macaque* CCHF animal models have allowed significant advancements in vaccine development.^75–80^ The impact of type I and/or type II interferon deficiency on CCHF vaccine-induced adaptive immune responses, however, deserves further evaluation. The disease spectrum in the *Cynomolgus macaque* depicts disease states seen in humans.^79^ Despite issues around variability in observed disease outcomes,^81^ the cost, and size of the animals, this immunocompetent animal model will be valuable in the development of CCHFV therapies. The addition of a humanized mouse model, which previously exhibited strain-specific virulence by producing different disease outcomes by a CCHFV Turkish and an Oman strain^78^ would be a valuable addition to evaluate the interplay between pathogenicity and immunogenicity.

Currently, immune responses conferring protection following CCHFV infection have not yet been described. CCHFV-neutralizing antibodies are likely produced against the Gn and Gc glycoproteins, which bind target cells,^97^ thus vaccine attempts focused on the M segment. Studies have demonstrated the absence of correlation between *in vitro* neutralization and protection in the available mouse models.^89,90,98,100^ Future studies should probe and delineate factors responsible for the observed differences between *in vitro* neutralization and protective efficacy. CCHFV is genetically diverse and the concern is whether a single vaccine can protect against global CCHFV strains. So far, efficacy studies against genetic strains have only been investigated for a VRP vaccine candidate.^110^ Recently, NP-based vaccine candidates have also resulted in complete protection in knockout mice despite the absence of *in vitro* neutralizing antibodies.^89,90^ The NP has thus proved to be an important vaccine target. Since B and T cell epitopes have been mapped on the glycoproteins and NPs, efforts can be directed in developing multiepitope-based vaccines. Epitope-based vaccines would be one way to develop an effective vaccine against the diverse CCHFV strains by selecting multiple antigenic epitopes. The design of epitope-based vaccines allows B or cytotoxic T lymphocyte (CTL) epitopes to be linked together in series with helper T lymphocyte epitopes ensuring CD4 T cells and pathogen-derived molecules are appropriately primed facilitating robust humoral and CTL responses.^119^ One characteristic of an ideal vaccine is that it should confer long-term sterilizing immunity after single administration. Despite several vaccine strategies providing complete protection of knockout mice after viral challenge (Table 1), protective single-dose regimens have been achieved by a rVSV-based vaccine expressing the GPC^116^ and a VRP vaccine,^109,110^ whereas the rest were administered using a prime/boost approach. Additionally, mice administered with the VRP vaccine did not develop clinical disease signs and CCHFV RNA was not detected in tissues at study end point. While complete protection has been achieved using a single-dose regimen, none of the available candidate vaccines has been evaluated for their ability to induce long-term immunity. Immune responses against either the NP or the GPC have been protective in efficacy studies necessitating the investigation of the role of both proteins in vaccine development. A thorough dissection of immune responses generated by the NP and GPC whether singly or in combination can help unmask the immune correlates of protection.

The use of genetic adjuvants to enhance immune responses in CCHF vaccine development has been sparsely investigated. Plasmid-expressing cytokines such as interferon-γ, interleukin (IL)-2, IL-12, Granulocyte/macrophage colony-stimulating factor,^120–122^ chemokines MIP-1α and RANTES,^123,124^ and ICAM-1, CD40L, and CD80/86 costimulatory molecules,^125–127^ have been investigated as genetic adjuvants *in vivo* with promising results in different settings. In the sole CCHF vaccine study using genetic adjuvants described in the literature, the CD24 costimulatory molecule was codelivered with the CCHFV NP. CD24 enhanced antibody and cytokine responses, although this was not translated to protective efficacy studies.^89^

The prototype IbAr 10200 CCHFV strain has mostly been used in vaccine studies. This prototype CCHFV strain was discovered in a tick^128^ and its virulence in humans is unknown. The NP from IbAr 10200 and AP92 strains did not antagonize interferon response *in vitro* as did the Hoti strain.^129^ In a study by Zivcec and colleagues,^130^ VLP-bearing glycoproteins from the IbAr 10200 CCHF strain displayed the reduced capacity to enter monocyte-derived macrophages. The effect in stimulating immune responses although remains to be elucidated. Considering the diversity of the CCHFV glycoproteins, it will be interesting to evaluate if differences in amino acid sequences between various global CCHFV strains does affect immunogenicity.

The utility of a BoHV-4 viral vector in comparison to an AdV-5 and a DNA vector in NP-based vaccine development was evaluated.^90^ BoHV-4 persists in monocytes and macrophages. Persistence in white blood cells could eliminate the need for booster doses for antigens delivered by BoHV-4. Besides that, delivering antigens by the BoHV-4 vector can enhance antigen presentation since the virus persists in monocytes and macrophages, which are antigen-presenting cells.^90^ Even though similar protection rates in knockout mice were obtained with vaccine constructs delivered with the BoHV-4 and AdV-5 vectors, the advantages offered by the BoHV-4 vector needs to be further explored in detail. The role played by the various expression systems in shaping CCHFV immune responses in animal models warrant investigation.

Since animals, particularly livestock, play an important role in CCHFV transmission cycle, the ability of vaccines to prevent viremia in livestock could reduce the rate of transmission to humans. CCHF human vaccine development has accelerated in recent years, and some vaccine studies have reported promising results in animal models. Whether these results can be translated to human clinical trials remains to be seen. Vaccine design and efficacy can be further enhanced by the delineation of correlates of CCHF protection which up to now have remained an enigma.

## Abbreviations Used

AdV-5: adenovirus type 5
BoHV-4: bovine herpesvirus type 4
BSL-4: biosafety level 4
CCHF: Crimean–Congo hemorrhagic fever
CCHFV: Crimean–Congo hemorrhagic fever virus
CD24: cluster differentiation 24
CHF: Crimean hemorrhagic fever
CTL: cytotoxic T lymphocyte
g.g.: gene gun
GPC: glycoprotein precursor
i.d.: intradermal
i.m.: intramuscular
i.n.: intranasal
IP: intraperitoneal
IS: transiently suppressed type 1 interferon system
mRNA: messenger RNA
MVA: modified Vaccinia Ankara virus
NP: nucleoprotein
rVSV: recombinant vesicular stomatitis virus
s.c.: subcutaneous
SNPs: single nucleotide polymorphisms
Th: T-helper
USSR: Union of Soviet Socialist Republics
VLP: virus-like particle
VRP: virus-like replicon particle
